# Reply to Salimi, M. Comment on “Patsaki et al. Benefits from Incorporating Virtual Reality in Pulmonary Rehabilitation of COPD Patients: A Systematic Review and Meta-Analysis. *Adv. Respir. Med.* 2023, *91*, 324–336”

**DOI:** 10.3390/arm92010004

**Published:** 2023-12-22

**Authors:** Irini Patsaki, Vasiliki Avgeri, Theodora Rigoulia, Theodoros Zekis, George A. Koumantakis, Eirini Grammatopoulou

**Affiliations:** Department of Physiotherapy, University of West Attica, 12243 Athens, Greeceigrammat@uniwa.gr (E.G.)

We are writing in response to the comment [1] send by Mr. Mostafa Salimi regarding our study “Benefits from Incorporating Virtual Reality in Pulmonary Rehabilitation of COPD Patients: A Systematic Review and Meta-Analysis” [2] that was published in the journal “Advances in Respiratory Medicine”. Looking into the data included in the meta-analysis that were extracted by Suntanto et al. [3], we did mistakenly enter the control group data in place of the experimental group data, and vice versa, as Mr. Salimi noted. We re-examined all data extracted from all studies, and we found that this mistake was limited to the study by Suntanto et al. [3]. We ran the meta-analysis again for both the outcomes analyzed that included this study (6MWT and MRC dyspnea). The Review Manager software (RevMan v.5.4.1) was used to summarize the effects of VR training on exercise capacity (6-Minute Walk Test—6MWT) and subjective feeling of dyspnea (Medical Research Council Scale—MRC). With regard to 6MWT, we found that there was a marginally non-statistically significant effect (*p* = 0.05). More specifically, a mean difference [MD] (95% CI) = 15.93 (−0.14 to 31.99) m, favoring VR training, with marginally non-statistical significance (Z = 1.94, *p* = 0.05) and statistical heterogeneity (I^2^ = 72, *p* = 0.01), was noted. With regard to dyspnea, we again found a positive effect but no statistical significance. More specifically, we found a mean difference [MD] (95% CI) = −0.15 (−0.45 to 0.15), with both studies favoring VR training but, overall, not reporting statistical significance (Z = 1.00, *p* = 0.31), with no statistical heterogeneity (I^2^ = 0, *p* = 0.74). Mr. Salimi referred to a meta-analysis on quality of life, but this was not performed and not presented in our article. We would like to thank Mr. Salimi for noticing this mistake and giving us the opportunity to make all the appropriate corrections in order to reassure readers of the scientific accuracy of our findings. Virtual reality is a very promising technology in rehabilitation, and this systematic review and meta-analysis provides important information not only regarding clinical implementation but also for further research.
